# Neural and Behavioral Effects of a Novel Mu Opioid Receptor Antagonist in Binge-Eating Obese People

**DOI:** 10.1016/j.biopsych.2012.10.022

**Published:** 2013-05-01

**Authors:** Victoria C. Cambridge, Hisham Ziauddeen, Pradeep J. Nathan, Naresh Subramaniam, Chris Dodds, Samuel R. Chamberlain, Annelize Koch, Kay Maltby, Andrew L. Skeggs, Antonella Napolitano, I. Sadaf Farooqi, Edward T. Bullmore, Paul C. Fletcher

**Affiliations:** a Department of Psychiatry, Behavioural & Clinical Neuroscience Institute, Cambridge Biomedical Campus, University of Cambridge, United Kingdom; b Medicines Discovery and Development, GlaxoSmithKline, Clinical Unit Cambridge, Addenbrooke’s Centre for Clinical Investigations, Cambridge, United Kingdom; c Academic Discovery Performance Unit, GlaxoSmithKline, Addenbrooke’s Centre for Clinical Investigation, Cambridge, United Kingdom; d Metabolic Research Laboratories, Institute of Metabolic Science, University of Cambridge, Cambridge, United Kingdom; e Cambridgeshire and Peterborough National Health Service Foundation Trust, Cambridge, United Kingdom

**Keywords:** Binge eating, fMRI, hedonics, motivation, obesity, opioid

## Abstract

**Background:**

Binge eating is associated with obesity and has been conceptualized as “food addiction.” However, this view has received only inconsistent support in humans, and limited evidence relates key neurocircuitry to the disorder. Moreover, relatively few studies have used pharmacologic functional magnetic resonance imaging to probe the underlying basis of altered eating behaviors.

**Methods:**

In a double-blind, placebo-controlled, parallel group study, we explored the effects of a potent mu-opioid receptor antagonist, GSK1521498, in obese individuals with moderate binge eating. Subjects were tested during a baseline placebo run-in period and retested after 28-days of drug (*n* = 21) or placebo (*n* = 21) treatment. Using functional magnetic resonance imaging and behavioral measures, we determined the drug’s effects on brain responses to food images and, separately, on motivation to expend energy to view comparable images.

**Results:**

Compared with placebo, GSK1521498 was associated with a significant reduction in pallidum/putamen responses to pictures of high-calorie food and a reduction in motivation to view images of high-calorie food. Intriguingly, although motivational responding was reduced, subjective liking for the same images actually increased following drug treatment.

**Conclusions:**

Stimulus-specific putamen/pallidal responses in obese people with binge eating are sensitive to altered mu-opioid function. This neuromodulation was accompanied by reductions in motivational responding, as measured by grip force, although subjective liking responses to the same stimuli actually increased. As well as providing evidence for a link between the opioid system and food-related behavior in binge-eating obese individuals, these results support a dissociation across measures of motivation and liking associated with food-related stimuli in these individuals.

Human and animal studies of reward processing demonstrate that motivation towards obtaining, and the hedonic value of a reward, though highly related, are dissociable. This has been framed as a dissociation between “wanting” and “liking,” subserved by dopaminergic and opioidergic systems respectively (1). Although this perspective has generated debate and it has been pointed out that the dopamine-wanting perspective resonates strongly with a previously expressed view of dopamine’s role in the “activation” of behaviors (2), there is a broad consensus that behaviors may be highly motivated toward the acquisition of outcomes even when those outcomes have limited hedonic value, as in habitual responding (3). This important observation is a cornerstone of models of addiction (4,5), in which a critical element of the addictive process is the transition to habitual behavior that is relatively insensitive to the current hedonic value of the outcome.

A key mediator in the hedonic valuation process is the mu-opioid receptor (MOR) system (6–12). In humans, mu-opioid antagonists reduce the hedonic responses to, and consumption of, palatable foods. The system also has a role in the motivational aspects of food-related behaviors (13), mediated by interactions with dopaminergic systems (14). MORs localized on inhibitory gamma-aminobutyric acid-ergic interneurons in the ventral tegmental area (VTA) and hypothalamus can modulate dopamine release in the nucleus accumbens and other dopaminergic target areas (15–17). Furthermore, MOR knockout mice demonstrate decreased firing frequency (including reduced bursting activity) of midbrain dopamine neurons (18) and decreased dopamine reuptake in the nucleus accumbens (19). The system has also been implicated in animal models of binge eating, with MOR antagonism reducing such behaviour (13,20,21). Although the effects are less clear in humans, there is some genetic evidence that implicates the gain of function 118G polymorphism of *OPRM1*, the MOR gene, in binge eating disorder (22).

It has been argued that a food addiction process is relevant to the development of obesity, particularly in those who binge (23,24). However the evidence supporting this in humans has been questioned on clinical/behavioral (25) and neuroscientific (26) grounds. A critical challenge in furthering the neuroscientific exploration of this issue is establishing the functional neuroanatomy and neurochemistry of the systems that subserve motivation toward and enjoyment of foods and how they may be perturbed in conditions such as binge eating. Pharmacologic imaging studies offer a powerful way of characterizing these systems and may offer insights that suggest ways of identifying, developing, and refining therapeutic strategies (13,27,28).

Previous pharmacologic imaging studies in obesity (29–35) have focused largely on serotonergic and dopaminergic mechanisms; opioid mechanisms have received little investigation. Given their critical role in food reward processing and potentially in binge eating, as well as their implication in substance addictions (36), further investigation of the MOR is warranted, both as a potential pharmacologic target and as a neural system relevant to the understanding of normal and aberrant eating behavior. This study sought to examine this system using GSK1521498, a potent antagonist with 14- to 20-fold selectivity for MORs, in otherwise healthy, obese volunteers who were moderate binge eaters. The aim was to determine, in the context of a clinical trial of this drug’s effects on weight and eating behavior, the concurrent changes in behavioral and neural responses to food images in obese individuals with a target behavior potentially sensitive to this receptor modulation.

In both preclinical models of obesity and binge eating (21,37) and a Phase 1 study in healthy overweight humans (38), GSK1521498 has been shown to reduce food intake, particularly of high fat/sugar foods. These findings were supported in this 28-day treatment trial (39) in which treatment with GSK1521498 5 mg/day led to a significant reduction in hedonic responses to high fat and high sugar foods and a reduction in ad libitum consumption, particularly of high fat foods. However, no overall weight loss was found. Furthermore, in animal models, GSK1521498 has been shown to reduce food seeking (21).

The tasks reported here were chosen to explore drug-related changes in motivation and pleasure associated with food stimuli. During functional magnetic resonance imaging (fMRI), subjects viewed images of high and low calorie foods and high and low reward nonfoods, making liking responses for each. In a novel, complementary behavioral task outside the scanner, we measured the effort volunteers were willing to expend (on a grip force transducer) to view images of different types and whether drug treatment affected this effort measure and subjectively rated “liking” responses.

## Methods and Materials

Sixty-three volunteers (28 [44%] males) aged 18 to 60 years (mean±SD, 41.5±10.0 years) endorsing moderate-severe binge eating (Binge Eating Scale [BES] scores≥19) (40,41) (mean 26.4±6.7), and with body mass index≥30 kg/m^2^ (mean 37.3±4.76 kg/m^2^), were enrolled in this study. The study (identification number EudraCT 2009-016663-11, ClinicalTrials.gov identifier NCT01195792) was approved by Berkshire Research Ethics Committee (United Kingdom), and all participants provided signed informed consent.

### Study Design

**Figure 1:**
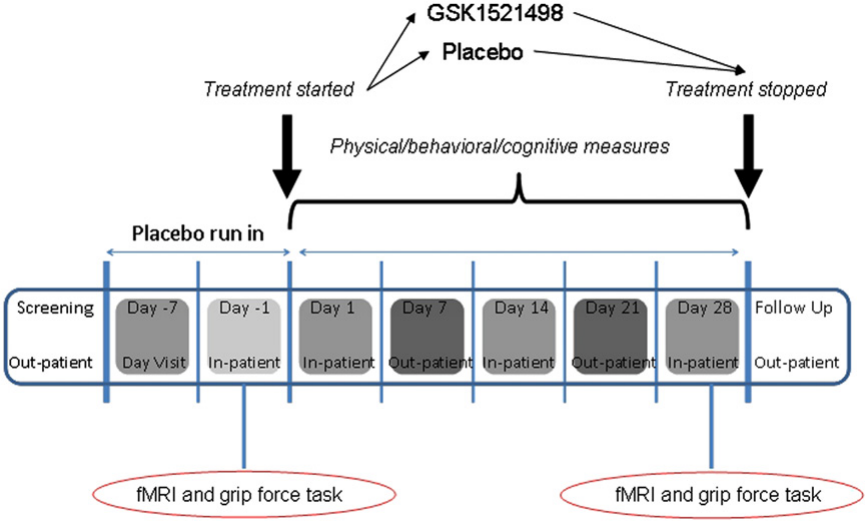
Study design. Following screening and a 7-day single-blind, placebo run-in, a baseline assessment was performed during a 2-day stay on the clinical research unit. This included the functional magnetic resonance imaging (fMRI) and grip force tasks that are the focus of this article. Thereafter, participants were randomised to receive oral GSK1521498 (5 mg) or placebo for 28 days. Further inpatient assessments were performed on Day 14 and Day 28. fMRI and grip force task were repeated on Day 28. Several other physical, cognitive, and eating measures were performed over the course of the visits (see Ziauddeen *et al*. [26] for details).

In a double-blind, placebo-controlled, parallel-group design, subjects received 1-week single-blind placebo run-in, followed by 4-week treatment with either placebo (*n* = 21), GSK1521498 2 mg/day (*n* = 21) or GSK1521498 5 mg/day (*n* = 21). Full details of the study are reported elsewhere (39) and summarized in Figure 1. On Day 1 (predrug), each participant underwent fMRI scanning and the behavioral and eating measures described subsequently. Following 28 days of treatment, subjects returned for full evaluation and repeated all measures. Here we consider only data from the placebo and GSK1521498 5 mg/day groups. The 5-mg dose achieved 82% to 92% 24-hour MOR occupancy compared with 64% to 80% with the 2-mg dose, which showed no effect on eating behavior (39). Thus, data from the 2-mg group were not analyzed here to minimize multiple comparisons conducted in investigating drug effects at a dose unlikely to have a pharmacodynamic effect.

**fMRI Task.** Participants fasted for 10 to 12 hours and performed a simple task adapted from one reported previously (34,42). The task entailed viewing and reporting subjective liking for images from four categories: high-calorie foods (e.g., chocolate), low-calorie foods (e.g., broccoli), rewarding nonfood items (e.g., watches, jewelry) and less rewarding nonfood items (e.g., staplers). Images were matched across categories for color, size, and background. Thirty images from each category were presented in blocks of 5, resulting in a total of 120 images over 24 blocks. Each image was presented for 4 sec with a 1-sec intertrial interval (block length = 22 sec). Image blocks were randomly interspersed with fixation periods. Participants were instructed to press a button to indicate their liking for each image with duration of button press indicating their rating. A mixed-effects analysis of variance (ANOVA) model was used for the behavioral analysis.

**Image Acquisition and Analysis.** Because of an initial problem with task randomization, imaging data were unreliable for the first three participants. Eight participants could not undergo both scanning sessions, and one could not fit into the scanner. We therefore confine our analysis to the 30 reliable datasets: 14 placebo (mean age 40.6±8.5 years, 6 men) and 16 drug (mean age 39.8±10.2 years, 8 men). Details on image acquisition and preprocessing are in Supplement 1.

A two-stage masking procedure was used to maximize sensitivity while minimizing the multiple comparisons problem. First, an overall set of regions of interest (ROIs) was a priori defined using the PickAtlas tool (43) implemented in SPM5 (Wellcome Trust Centre for Neuroimaging, London, United Kingdom) and consisted of the following bilaterally symmetric ROIs: nucleus accumbens, caudate nucleus, putamen, globus pallidus, midbrain, prefrontal cortex, insula, hypothalamus, and amygdala. This mask was further refined and constrained using data from all participants combined at Day –1 (i.e., pretreatment) for the analyses relating to the main effects of image type (all food vs. nonfood and high-calorie vs. low-calorie images). This combined anatomic/functional mask was used to test drug effects for statistical significance while maintaining a family-wise error (FWE) correction for multiple comparisons entailed by voxelwise testing of the entire set of regions of interest.

**Identifying Regional Responses and Drug Modulations of Key Conditions.** Initial analysis and defining the mask: the purpose of the initial analysis was twofold: first to ensure that across all individuals at baseline, the key task manipulation, notably viewing high-compared with low-calorie food images, produced activation in expected brain regions. This analysis was restricted to the set of ROIs described earlier, and FWE correction (*p*<.05) was used. Second, to identify brain regions in which the response to high reward was specific to food images, another analysis was conducted assessing the interaction of stimulus type (food and nonfood) with reward value (high- vs. low-calorie food and high- versus low-reward nonfood).

Analysis of drug effects: The mask defined earlier was used in all the following analyses to characterize the effects of GSK1521498 compared with placebo at Day 28. Three key contrasts were tested:

1. Food type by drug interaction (Day 28 data). This analysis identified regions in which there was a greater activation for high-calorie compared with low-calorie food stimuli in the placebo group compared to the GSK1521498 group, that is, regions in which GSK1521498 attenuated brain activation to high-calorie food images specifically.
2. Stimulus type by reward by drug interaction (Day 28 data). To determine whether any observed effect was specific to food, the nonfood (high reward vs. low reward) conditions were included in the analysis. This, of course, is not independent of the foregoing analysis but was done for completeness.
3. Food type by drug by time (Day –1 and Day 28 data). Here we examined the baseline and posttreatment scans for both groups to assess the drug effects in terms of a change from the baseline. Given that at this baseline, all subjects were at the end of a 7-day placebo run-in, this analysis is presented for completeness but with due caution.

Correlations of drug-related alteration in fMRI signal with other measures: for these secondary analyses, parameter estimates were extracted from any region(s) showing a task-specific drug effect. We sought to determine whether variability in weight change, ad libitum buffet meal intake (39) (Supplement 1), BES scores, and effort expended on the grip force task were related to neural changes in produced by GSK1521498 in the drug group.

**Grip Force Task.** The purpose of this task was to examine the physical effort participants were willing to expend to view specific images and to relate this to their subsequent subjective liking ratings of the images. Participants were seated approximately 60 cm from a computer screen and held a grip force transducer (GFT) in one hand. The isometric GFT (TSD121C, Biopac, Goleta, California) was connected to a Biopac MP150 unit through a Biopac DA100C module. This module was interfaced with MATLAB (Mathworks, Natick, Massachusetts) and Cogent (Laboratory of Neurobiology, University College of London) on a standard laptop.

The task comprised 216 trials, each lasting 5 sec. On each trial, two images appeared side by side onscreen: one large (300×300 pixels**)** and clearly visible (the default image), the other very small (5×5 pixels**)** with the image content indeterminable (nondefault image). Exerting force on the GFT would proportionately increase the size of the nondefault image and shrink the default image. The force required to maximally increase the size of the nondefault image was set at 10% of each individual’s maximum grip force as calibrated at the start of the task. Participants were instructed that they could view images as they chose by squeezing more or less on the grip force transducer, and were left alone for the duration of the task.

Three categories of images were used: high-calorie food, low-calorie food, and rewarding nonfood (e.g., jewelry, electronic gadgets depending on gender). Each image pair comprised an image from each of two categories. Every image was paired with all images from the other two categories. The pairs were counterbalanced with respect to the starting position on the computer screen such that each image was presented on an equal number of trials as the default and nondefault image. Therefore, for each image, there were an equal number of trials in which expenditure of effort would enlarge it or reduce it. Finally participants rated their liking for each image on a visual analogue scale (0-100 mm).

**Analysis.** The force-time curves were transformed into a summary measure for each image by averaging the area under the curves across trials in which it was the nondefault image (i.e., when effort would bring it to the foreground). Summary force and liking measures were generated for each image category for each subject. The change from baseline for both force and liking measures were taken to a group-level ANOVA with treatment group and category as factors. We also explored correlations between grip force and liking scores and other pharmacodynamic endpoints.

## Results

### fMRI Task

**Figure 2:**
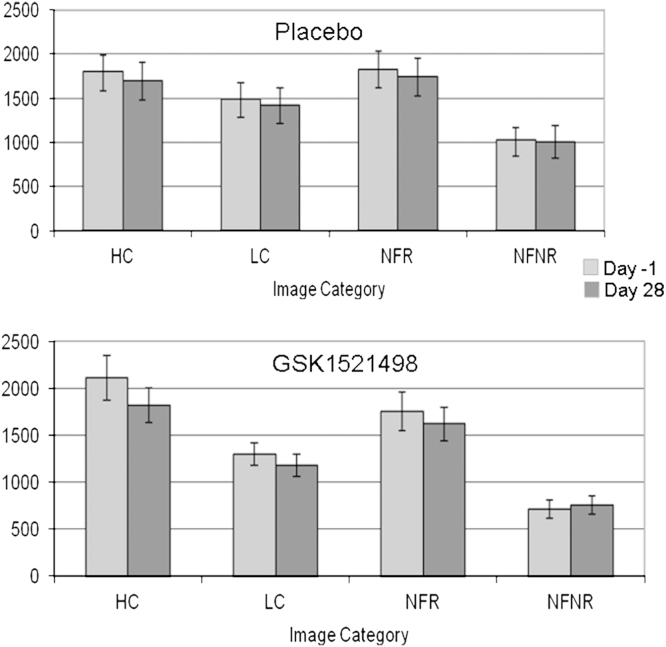
Functional magnetic resonance imaging task: liking ratings. Subjective liking of food images was assessed (see Methods and Materials) using duration of button press and is represented here in milliseconds. Average values for each of the four image categories are shown before (Day –1) and after (Day 28) drug and placebo treatment. HC, high-calorie images; LC, low calorie images; NFR, nonfood rewarding images; NFNR, nonfood, nonrewarding images.

**Behavioral Analysis.** There was an overall effect of image type, with higher liking ratings for food compared with nonfood images [*F*(1,13) = 10.1, *p*<.01; Figure 2]. There was also a main effect of reward level with greater liking ratings for high-calorie food and high-reward nonfood images [*F*(1,13) = 34.7, *p*<.001]. In addition, there was a significant interaction between the reward level and the image type with a greater difference in liking ratings between high- and low-reward nonfood images than between high- and low-calorie food images [*F*(1,13) = 6.9, *p*<.05]. No other main effects or interactions were significant, and no significant drug effects were found.

**Figure 3:**
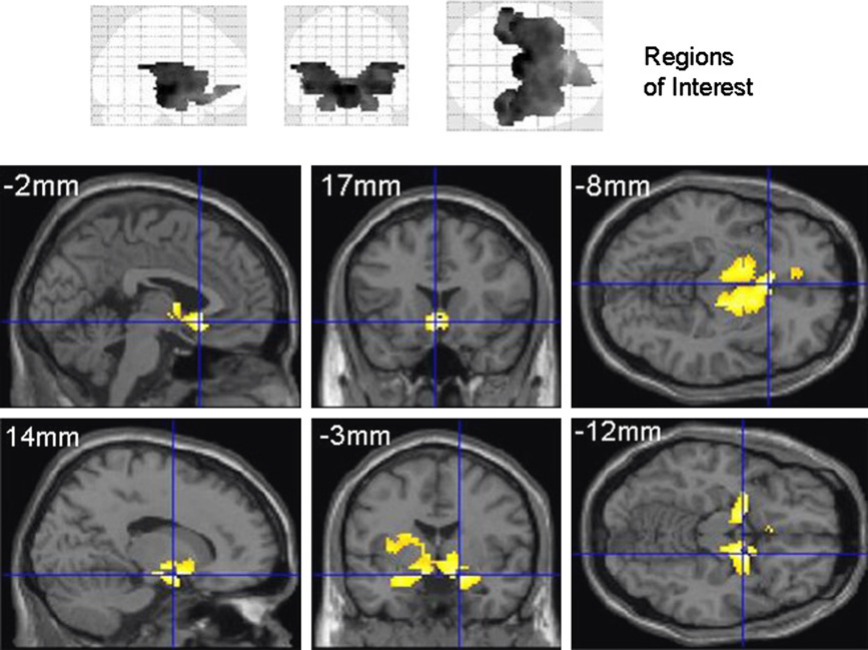
Functional magnetic resonance imaging results at Day –1. The upper panel depicts the regions of interest used on orthogonal maximum intensity projections (“glass brains” viewed from the right (left projection), from behind (middle) and from above (right). The lower panels show the significant (*p*<.05, uncorrected for display) areas of activation when contrasting viewing of high-calorie to low-calorie food images at Day –1. This contrast used the placebo and GSK1521498 treatment groups combined and shows activity across key regions of reward circuitry. Full details of the activation foci are presented in Table 1. Areas of significant activation are rendered onto a standard template image in Montreal Neurological Institute space with sections chosen at the coordinates most appropriate to display the key activations. This activation map was used as a mask to constrain subsequent analysis exploring condition-specific effects of GSK1521498.

**Imaging Analysis.** Initial analysis (Day –1; Figure 3 and Table 1). A contrast across food images (high calorie>low calorie) on baseline data (Day –1) using the mask of a priori ROIs evoked significant activation within a large set of these regions (Figure 3 and Table 1). As described, this analysis constrained all subsequent analyses.

**Figure 4:**
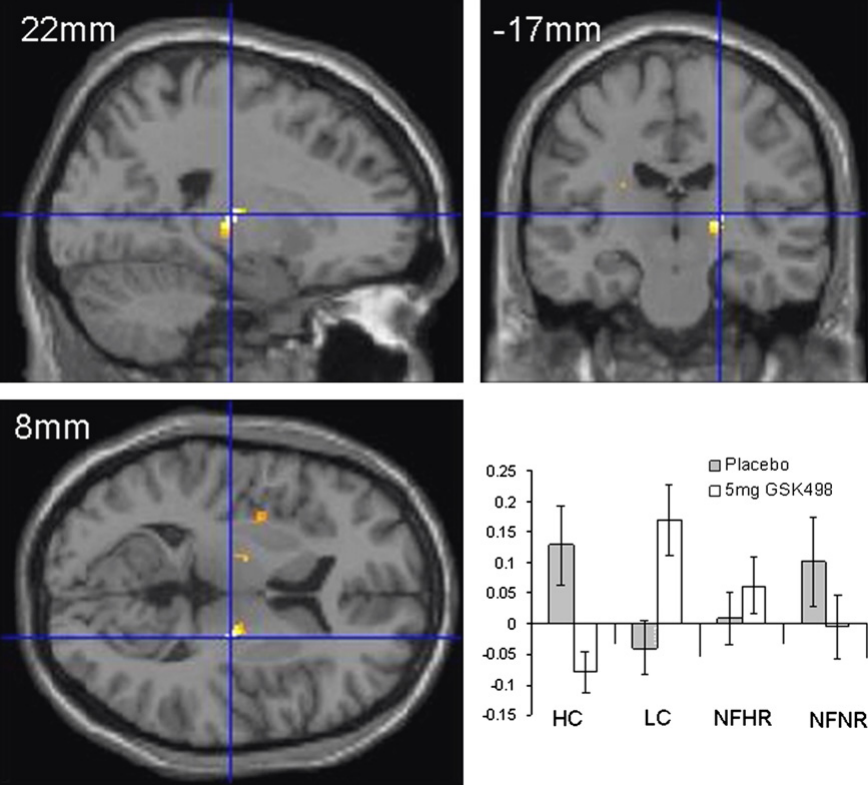
Functional magnetic resonance imaging (fMRI) results Day 28: drug-by-stimulus-by-reward interaction. fMRI results at Day 28 showing drug by stimulus type (food vs. nonfood) by reward type (high calorie/high reward vs. low calorie/low reward). The pallidum/putamen region demonstrating this interaction (*p*<.05, uncorrected for multiple comparisons for display) is shown superimposed onto orthogonal sections of a structural MRI in standard Montreal Neurological Institute space. In the lower right panel are plotted the parameter estimates for each of the stimulus types after placebo and GSK1521498 treatment. HC, high-calorie images; LC, low calorie images; NFHR, nonfood high reward images; NFNR, nonfood, nonrewarding images.

Effects of GSK1521498 on brain responses to food stimuli:

1. Food type by drug interaction (Day 28 data). Right putamen/pallidum (x, y, z = 22, –17, 8; *Z* score = 3.7; *p*<.05 FWE corrected for the ROI mask) was the only region showing a significant attenuation of the neural response to high-calorie food images (compared with low calorie) by GSK1521498.
2. Stimulus type by reward by drug interaction (Day 28 data). A significant effect was observed in the right putamen/pallidum (x, y, z = 22, –17, 8; *Z* score = 3.7; *p*<.05 FWE), demonstrating that the drug effect on high calorie versus low calorie images was greater than on high reward versus low reward nonfood images (see Figure 4).
3. Food type by drug by time. This analysis explored time-dependent drug effects on high-calorie versus low-calorie stimuli. Although there was a strong trend for a food type by drug by time interaction in the same region of right putamen/pallidum (x, y, z = 22, –19, 8; *Z* score = 2.7), this did not survive correction for multiple comparisons within the prespecified mask.

Correlations between fMRI and behavioral measures: several correlation analyses were carried out to explore the relationship between drug effects on imaging, weight change, BES scores, and eating behavior. Because these findings did not survive multiple comparisons corrections, we treat these as preliminary and speculative and do not discuss them further. In brief, there was a correlation between drug-induced modulation of pallidal/putamen activity and weight change (Spearman’s rho = .46, one-tailed *p* = .038) and a trend toward correlation with reduction in consumption of 60% fat dessert **(**Spearman’s rho = .49, one-tailed *p* = .055)

**Figure 5:**
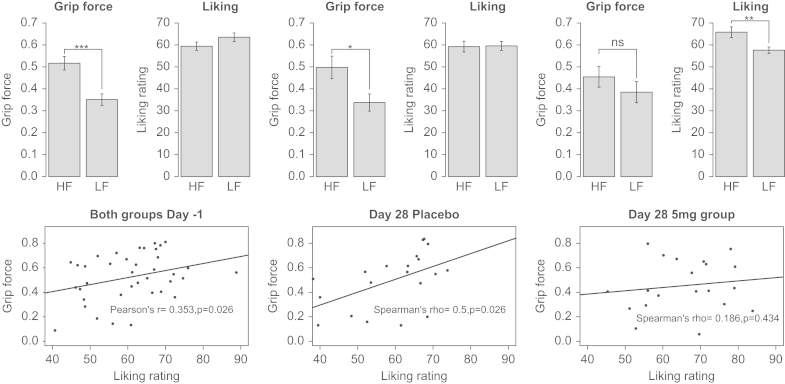
Grip force task: effort expended and liking ratings. The top panel shows the grip force exerted and the corresponding liking ratings at baseline in both groups and separately for placebo and the GSK1521498 group at the end of treatment. It can be seen that the difference between force exerted for high-fat (HF) versus low-fat (LF) food images is no longer significant after drug treatment, even though the subjective liking for these images is higher. The bottom panel displays the correlations between the exerted force and liking ratings for the high-fat food images, again at baseline for both groups and separately at Day 28. The correlation between these two measures is seen at baseline and in the placebo group at the end of treatment but is lost in the drug group. *p < .05; **p < .01; ***p < .001.

**Figure 6:**
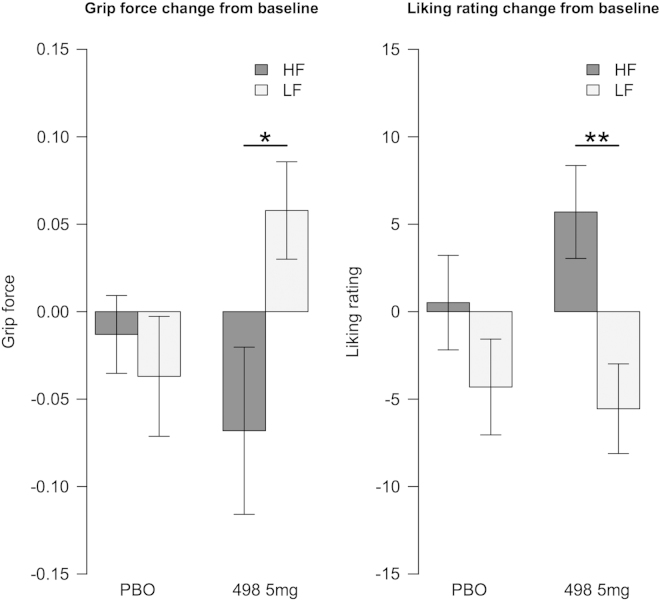
Change from baseline in grip force and liking ratings. *p < .05; **p < .01. HF, high fat; LF, low fat; PBO, placebo.

**Grip Force Task.** Complete data were available for 40 participants. One participant from each group was excluded from the analysis because no baseline data were available. No significant effect of drug group or visit was seen for the nonfood images (Table S1 and Figure S1 in Supplement 1), so subsequent analyses are presented only for food images. On Day –1, both groups were on placebo and were pooled for the baseline analysis, there being no significant group differences (force: *F* = .1925, *p* = .66, liking: *F* = .0159, *p* = .9). Grip force for high-calorie images was significantly greater than for low-calorie images (Figure 5, top panel). Although this effect persisted after 28 days in the placebo group, it was no longer significant following treatment with GSK1521498. When the change from baseline (Figure 6) was examined, a significant group-by-food-category interaction (*F* = 4.753, *p* = .032) was observed. Thus, 5-mg GSK1521498 significantly attenuated the tendency to exert a greater grip to view high-calorie compared with low-calorie images.

The liking ratings were not different for the high-fat images compared with the low-fat at baseline; mean ratings for both were moderately high, although this is at odds with the expended effort. There was a significant effect of category in the ANOVA of change from baseline scores (*F* = 9.088, *p* = .0035). This was driven by the drug group, whose liking ratings increased for the high-fat food images (*p* = .0042), whereas their expended effort decreased (Figure 5, top panel); the placebo group showed the same pattern as at baseline.

Correlations were examined between grip force and subsequent liking rating. In brief, there was a significant positive correlation between grip force and liking at baseline for the high-calorie food images. This persisted in the placebo group on Day 28 but was no longer present following treatment with GSK1521498 (details in Figure 5, bottom panel).

## Discussion

We examined the neural and behavioral responses to 28 days of treatment with a potent MOR antagonist in obese people with moderate binge eating. We demonstrate two key findings. First, GSK1521498 produced a reduction in right pallidum/putamen responses to high-calorie food images despite no effect on subjective liking of the images. Second, although it reduced effort expended to view high-calorie food images, there was, unexpectedly, an increase in subjective liking ratings of those same high-calorie foods. Moreover, although the motivational measure correlated strongly with liking ratings before treatment, this relationship was lost after drug administration. These findings complement a previous report (39) and suggest that, in this group of obese people, the drug’s neural effects are associated with a potentially therapeutic perturbation in the relationship between motivation and subjectively experienced pleasure for the high-calorie food images.

Before treatment, there was widespread activation in reward-related regions to high-calorie food images, consistent with previous studies (34,44,45). The drug’s impact was localized to a brain region central to the motivational and hedonic components of eating and has been postulated to be a final limbic outflow pathway from the reward system (46). Although we observed no impact of GSK1521498 on the liking ratings for these images, it was associated with an increase in the liking ratings for the images in the grip force task. This latter finding is most intriguing, especially given that GSK1521498 has a negative impact on the pleasures associated with tasting highly palatable foods (39,47). It appears that, following GSK1521498 treatment, although motivation (grip force) is reduced, the verbal expression of liking distinguished high-calorie from low-calorie food images, a pattern that was expected, but not found, at baseline.

Clearly there is a disconnection between the imaging and motivational measures and their respective liking measures. An important consideration is that, in both these tasks, it is not consummatory reward processing that is being examined. It may be that the motor expression of motivation toward food reflects a more implicit measure of participants’ attitudes toward these high-calorie foods whereas the subjective liking reflects a more explicit one influenced by other factors (e.g., the reluctance that an obese person might have for publically expressing strong liking for high-calorie foods or the contribution of a healthiness attribute to low-calorie foods). In trying to understand this, it is worth considering the precise localization of the drug’s effect (i.e., overlaying posterior globus pallidus and putamen). It is tempting to speculate that this drug modulation lies on the uppermost aspect of the globus pallidus, but the spatial resolution does not allow definitive localization. However given the clear evidence that the pallidum is the site of a “hedonic hotspot” (6,46) rich in MORs and is a key brain region subserving the hedonic-motivation interface, the attenuating effects of an antagonist in precisely this region strongly suggest that this may well be the pallidum. If so, the localization of the effect in this region with an accompanying decrease in motivational responding indicates that the effect of GSK1521498 is to disrupt motivational responding to food or “food seeking” in the absence of actual reward consumption. This is in keeping with the findings from animal models wherein GSK1521498 reduces food seeking even before the animal has first experienced the food after drug administration (21). In the same animal model, the drug also affected the amount of food consumed, a finding replicated in humans (39).

This difference between anticipatory/motivational processes and consummatory/hedonic processes may partly explain an important limitation of these findings: why, aside from nonsignificant correlations with weight change and high-fat dessert consumption, the neural and motivational changes associated with drug treatment did not predict behavioral changes. Another limitation to be considered is the lack of correlation between the neural and motivational measure, which may relate to methodologic differences in the two tasks, the imaging task featured single unique presentations of multiple images and the GFT featuring multiple presentations of a small set of images, each always in contrast with another image.

How do these findings relate to food addiction? Clearly, the exploration of opioid antagonist effects on motivational and hedonic processes draws heavily on concepts emerging from, or related to, the addiction literature. But do these findings support or refute an addiction model of overeating? Two important caveats must be stated. We did not have a non-binge-eating or nonobese control group in this study. Second, all participants were only moderate binge eaters. Bearing these in mind, our findings show that the MOR system is clearly implicated in motivational aspects of food reward, and these aspects can be dissociated from the liking/expected reward of the food. However the food addiction model, insofar as it has been described in neural processing terms, predicts a dissociation in the native state (i.e., the placebo condition) with enhanced anticipatory reward and reduced consummatory reward, or “wanting without liking” (48,49). Instead, we find that the placebo group shows greater motivated responding to foods they rate as highly liked, whereas treatment abolishes and perhaps even reverses this effect. Although these ideas must be treated as speculative, they do not support a food addiction model of overeating. Nevertheless the findings are important in two key regards. First, they add to the converging lines of evidence that indicate a role for the opioid system in binge eating. Second, they provide a critical element for evaluating cognitive neuroscientific models of abnormal eating, including addiction models, by elucidating an integral part of the functional neuroanatomical and neurochemical network that subserves these behaviors.

One additional, speculative point is that the neuromodulatory effects of GSK1521498 appear to entail, in part, an enhanced response to low calorie food images (see plot, Figure 4). Might the impact of the drug reflect an additional, enhanced motivation toward low-calorie foods? Although such an effect was not elucidated by our behavioral measures, it is noteworthy that dietary manipulations can lead to shifts in preference from high- to low-fat foods in humans (50) and rats (51).

In summary, our findings suggest that a key mechanism of action of GSK1521498 is a specific and significant reduction in motivation toward high-calorie foods. This is in keeping with a central role for the opioid system in food-related behavior in this population and points to its importance in driving consumption and overconsumption of high-calorie foods and the potential therapeutic relevance of MOR antagonists, such as GSK1521498, in mitigating such overconsumption.

## Figures and Tables

**Table 1:** Neural Responses at Day –1: High-Calorie Versus Low-Calorie Food Images

Region	Coordinates (x, y, z)	*Z* Score
Ventromedial Prefrontal	–2, 17, –8	4.4
	–10, 38, –7	3.1
Cortex/ Cingulate Gyrus	18, 42, –4	2.6
Amygdala		
Right	14, –3, –12	4
	16, –3, –12	4
Left	–14, –3, –12	3.8
	–28, –1, –15	3.8
	–20, –1, –12	3.5
Insula		
Right	28, –16, 21	2.9
	24, –32, 20	2.9
	38, –13, 21	2.8
Left	–26, –26, 22	3.4
	–36, –7, 15	3.1
Putamen/Pallidum		
Right	16, 1, 13	2.8
	24, 5, 15	2.7
	20, –11, 8	2.6
Left	8, –2, 2	3.4
	–22, –19, 6	3.9
	–18, –13, 8	3.4
	–32, –6, 6	3.3
Midbrain	–8, –12, –6	3.2
Accumbens		
Right	2, 11, –7	3.5
Left	–4, –14, –6	3.4
Hypothalamus	6, 3, –7	3.3

Regions demonstrating significant activation for high-calorie compared with low-calorie images. All survive small volume family-wise error correction for multiple comparisons.
