# Supplementary material for: Neural and Behavioral Effects of a Novel Mu Opioid Receptor Antagonist in Binge-Eating Obese People
Source: Biol Psychiatry. 2013 May 1;73(9):887–94. doi: 10.1016/j.biopsych.2012.10.022 (PMC3898083; doi:10.1016/j.biopsych.2012.10.022)
Supplement: Supplementary file 1 — Supplementary Material [file mmc1.pdf]

Neural and Behavioral Effects of a Novel Mu Opioid Receptor Antagonist in Binge-Eating Obese People

Supplemental Information

Supplemental Methods

In this section we describe the eating behavior measures and the pharmacokinetic parameters that were included in the correlational analyses.

Eating Behavior Measure: the Ad Libitum Buffet

An ad libitum buffet meal was used to examine eating behavior during the inpatient visits on Days -1, 14, and 28. At pre-specified times during the day, subjects indicated their preferences for the foods to be served at the dinner buffet, on a menu card. Familiar foods from local supermarkets, containing either 20%, 40% or 60% fat were used for the buffet but no information about fat content was provided. Equicaloric portions of all menu options were presented at the dinner buffet at approximately 6 pm for subjects to eat ad libitum for 60 minutes in a comfortable room and while viewing a pre-chosen entertainment program of neutral content. Approximately 6000 calories of food were presented at each buffet meal. The total calories consumed and the ranked preference of each item, were recorded.

Pharmacokinetic Parameters

For the estimation of pharmacokinetic parameters, plasma concentrations of GSK1521498 were sampled serially on days 1 and 28 at the following times: pre-dose and 1, 2, 3, 6, 8, 12, 14, and 24.5 hours post dose. Additional pre-dose samples were collected on days 7, 14 and 21 for the estimation of steady state (trough) concentrations. Human plasma samples were analyzed for GSK1521498 using a validated analytical method (1) based on protein precipitation, followed by high performance liquid chromatography - tandem mass spectrometry analysis. The lower limit of quantification for GSK1521498 was 0.1 ng/mL using a 50 µL aliquot of human plasma with a higher limit of quantification of 100 ng/mL. Quality control (QC) samples, prepared at 3 different analyte concentrations and stored with study samples, were analyzed with each batch of samples against separately prepared calibration standards. For the

analysis to be acceptable, no more than one-third of the total QC results and no more than one-half of the results from each concentration level were to deviate from the nominal concentration by more than 15%. The applicable analytical runs met all predefined run acceptance criteria.

Image Acquisition and Analysis

We used a 3 Tesla Siemens Trio scanner, with a 225 mm field of view at the Wolfson Brain Imaging Centre, Cambridge. 605 volumes were acquired using a T2*-weighted echo-planar imaging sequence with 36 slices covering the whole brain. Each slice was 3 mm thick with an interslice gap of 1 mm. Slices were interleaved, repetition time = 2100 ms, echo time; TE1 = 13 ms, TE2 = 31 ms, flip angle = 80˚, axial orientation = oblique and matrix size.

Data were analyzed using statistical parametric mapping in the SPM5 program (www.fil.ion.ucl.ac.uk). Images were realigned then spatially normalized to a standard template and spatially smoothed with an isotropic 3 dimensional Gaussian filter (8 mm full width at half-maximum). The time series in each session were high-pass filtered (with cut-off frequency 1/120 Hz) and serial autocorrelations were estimated using an AR(1) model.

Four experimental conditions were modelled using a box car function convolved with a canonical haemodynamic response: High Calorie (e.g. cake) and Low Calorie (e.g. salad) foods, Non-Food High Reward (e.g. mp3 player) and Non-Food Low Reward (e.g. stapler). Conditions were specified as covariates in a general linear model and the beta parameter estimated at each voxel for each stimulus type, was derived from the mean least-squares fit of the model to the data. The responses to each condition were compared to the fixation baseline, and each of these 4 these contrasts were taken forward to a group analysis treating inter-subject variability as a random effect.

Additional Whole Brain Analysis

While the imaging analysis focused on key regions of interest, defined anatomically and then functionally (see main text) we also, for completeness carried out a whole brain analysis of drug effects correcting for multiple comparisons across the entire search volume. No significant effects were found surviving this correction.

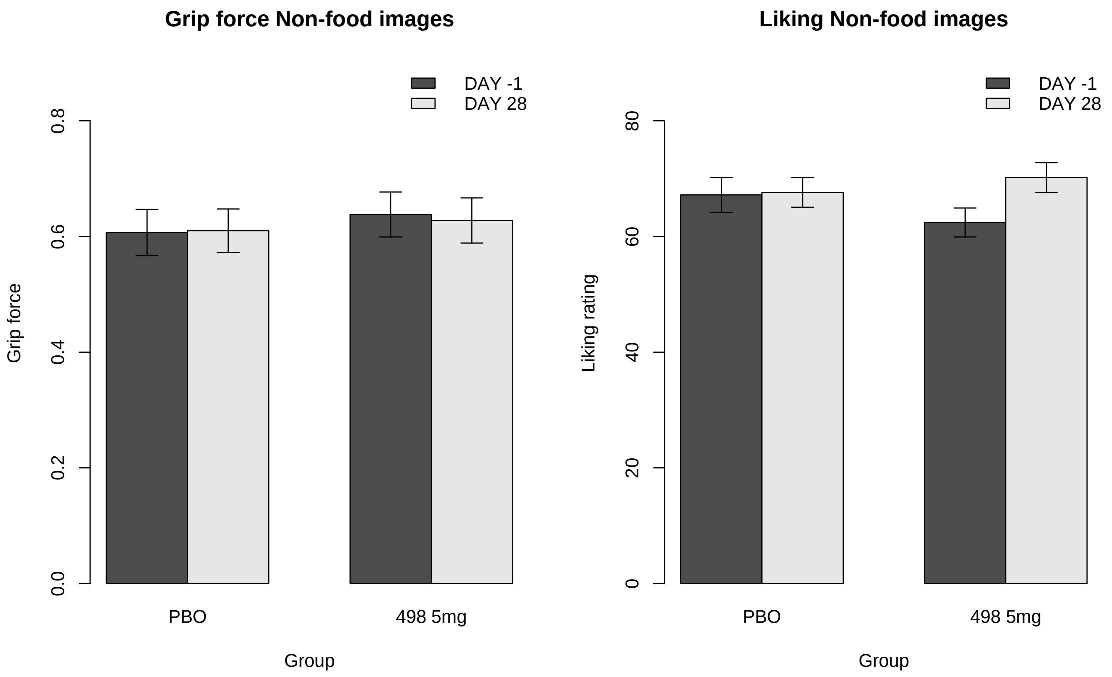

Figure S1. Picture surfing task: Comparison of non-food images across both visits and groups. There are no significant main effects or interactions between group and visit.

Table S1. Analyses of main effects and interactions for non-food images by group and visit

**Grip Force**					
	df	**Sum Sq**	**Mean Sq**	***F* value**	***p*-value**
Visit	1	0.00029	0.0002907	0.0096	0.9221
Group	1	0.01200	0.0120009	0.3970	0.5305
Visit:Group	1	0.00090	0.0009012	0.0298	0.8634
**Liking**					
	df	**Sum Sq**	**Mean Sq**	***F* value**	***p*-value**
Visit	1	337.6	337.57	2.3749	0.1275
Group	1	24.2	24.20	0.1703	0.6810
Visit:Group	1	266.5	266.45	1.8746	0.1750

Sq, square.

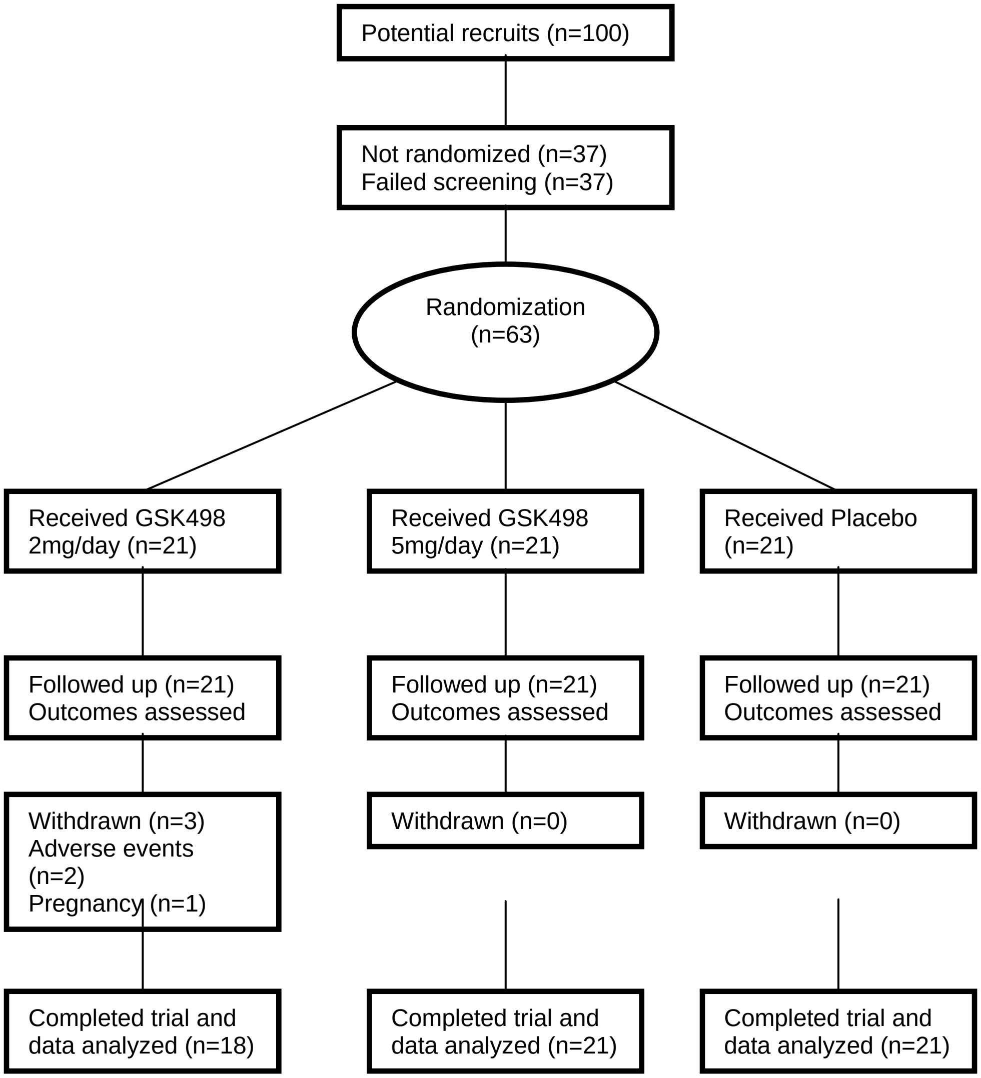

Figure S2. CONSORT diagram.

Supplemental Reference

1. Nathan PJ, O'Neill BV, Bush MA, Koch A, Tao WX, Maltby K, *et al.* (2012): Opioid receptor modulation of hedonic taste preference and food intake: A single-dose safety, pharmacokinetic, and pharmacodynamic investigation with GSK1521498, a novel {micro}-opioid receptor inverse agonist. *J Clin Pharmacol* 52:464-74.
